# Association between plasma glycocalyx component levels and poor prognosis in severe influenza type A (H1N1)

**DOI:** 10.1038/s41598-021-04146-2

**Published:** 2022-01-07

**Authors:** Xiao Huang, Feng Lu, Huanhuan Tian, Haoran Hu, Fangyu Ning, Quanmei Shang, Dong Hao, Weiwei Zhu, Guiqing Kong, Xiaohong Ma, Jiali Feng, Tao Wang, Xiaozhi Wang

**Affiliations:** 1 Department of Pulmonary and Critical Care Medicine and Intensive Care Unit, Binzhou Medical University Hospital, NO. 661 Huanghe 2nd Road, Binzhou, Shandong China; 2 Department of Oncology Internal Medicine, LinYi Cancer Hospital, Linyi, Shandong China; 3 Department of Pulmonary and Critical Care Medicine, Yantaishan Hospital, Yantai, Shandong China

**Keywords:** Predictive markers, Viral infection

## Abstract

Influenza A virus infection causes a series of diseases, but the factors associated with disease severity are not fully understood. Disruption of the endothelial glycocalyx contributes to acute lung injury in sepsis, but has not been well studied in H1N1 influenza. We aim to determine whether the plasma glycocalyx components levels are predictive of disease severity in H1N1 influenza. This prospective observational study included 53 patients with influenza A (H1N1) during the influenza season, and 30 healthy controls in our hospital. Patients were grouped by severity and survival. We collected clinical data and blood samples at admission. Inflammatory factors (tumor necrosis factor-α, interleukin-6, interleukin-10) and endothelial glycocalyx components (syndecan-1, hyaluronan, heparan sulfate) were measured. The plasma levels of syndecan-1, hyaluronan, and heparan sulfate were significantly higher in patients with severe influenza A (H1N1) than in mild cases. Syndecan-1 and hyaluronan were positively correlated with disease severity, which was indicated by the APACHE II and SOFA scores and lactate levels, and negatively correlated with albumin levels. At a cutoff point ≥ 173.9 ng/mL, syndecan-1 had a 81.3% sensitivity and 70.3% specificity for predicting of 28-day mortality. Kaplan*–*Meier analysis demonstrated a strong association between syndecan-1 levels and 28-day mortality (log-rank 11.04, *P* = 0.001). Elevated plasma levels of syndecan-1 has a potential role in systemic organ dysfunction and may be indicative of disease severity in patients with influenza A (H1N1).

## Introduction

Influenza is a viral disease, caused by infection with influenza A (or B) virus. Clinical symptoms range from mild to severe (including acute respiratory distress syndrome (ARDS))^1,2^. According to World Health Organization reports (http://www.who.int/mediacentre/factsheets/fs211/en/), influenza causes global annual infection rates of 5%*–*10% in adults, 3*–*5 million cases of severe illness, and between 290,000 and 650,000 deaths annually worldwide. Neuraminidase inhibitors (NAIs) are approved for both influenza A and B virus infections; early antiviral therapy with NAIs can improve the outcome of influenza A^2^; however, a small number of patients experience a rapid progression to primary viral pneumonia or secondary bacterial infections, resulting in a significant number of deaths despite the use of antivirals^3^. Therefore, improving antivirals alone may not be sufficient to minimize morbidity and mortality, and the pathogenesis of influenza A needs to be further explored.

Severe influenza virus infections cause a dysregulated inflammatory response, leading to the release of proinflammatory cytokines in the lungs and blood, a condition often referred to as a “cytokine storm”^4^. Inflammatory cytokines can lead to diffuse alveolar damage, and interstitial and airspace inflammation, which adversely affect outcomes in patients with severe influenza^5,6^. An abnormal immune response to influenza A can lead to endothelial damage, alteration of microvascular permeability, tissue edema, deregulation of coagulation, and even shock^7,8^.

Influenza A can directly and indirectly cause lung endothelial cell activation and injury, inducing microvascular leakage^9^. The surface of the vascular endothelium is coated with a “thick” glycocalyx, a dynamic and complex biochemical structure consisting of core proteins (syndecans and glypicans) and glycosaminoglycan chains (heparan sulfate [HS], hyaluronan [HA] etc.), which play a key role in limiting vascular permeability and regulating platelet and leukocyte adhesion^10^. Syndecan-1 (SDC-1) belongs to the family of syndecans, and is involved in leucocyte recruitment, the chemokine gradient, and extracellular matrix remodeling during inflammatory diseases^11^; it also plays a pivotal role in glycocalyx integrity and function. Glycocalyx dysfunction and component shedding have been described in many clinical pathophysiologic processes, including sepsis^12^, ARDS^13^, and coronavirus disease 2019 (COVID-19)^14^. Furthermore, elevated plasma SDC-1 and HA levels are associated with cumulative fluid volumes, degree of organ failure, and increased mortality in patients with sepsis^15,16^. Lipopolysaccharide induced shedding of HS in the pulmonary endothelial glycocalyx uncovers endothelial cell surface adhesion molecules, thereby accelerating neutrophil adhesion and alveolar exudation^17^. Benatti et al*.*^18^ reported that a higher level of HA and SDC-1 in flu syndromes with the ARDS group is observed compared to those without ARDS. These findings suggest that endothelial glycocalyx shedding occurs during the virus-induced ARDS installation. Thus, the role of endothelial glycocalyx injury and activation in the pathogenesis of ARDS caused by sepsis and flu is known, but the role of the endothelial glycocalyx in influenza A (H1N1) severity has not been elucidated. However, the effect of the components of the glycocalyx on the prediction disease severity of influenza A (H1N1) needs to be further investigated.

We further hypothesized that the degree of endothelial glycocalyx degradation is associated with severity and mortality in patients with influenza A(H1N1). In this study, we aimed to determine whether the plasma levels of glycocalyx components are indicative of influenza A (H1N1) severity.

## Materials and methods

### Study design and patient recruitment

This prospective observational study included patients admitted to the Department of Pulmonary and Critical Care Medicine (72 beds) and intensive care unit (48 beds) of Binzhou Medical University Hospital (Binzhou, Shandong, China) during a period of the influenza season from November 2017 to March 2018 and from November 2018 to March 2019. The inclusion criteria were the age of ≥ 18 years, and should be an influenza-positive PCR test from an airway specimen. Viral specimens were collected from the patients via nasopharyngeal swab, whereas lower airway specimens were obtained via endotracheal tube and bronchoalveolar lavage fluid. Influenza A (H1N1) reverse transcription PCR testing was performed in accordance with the Centers for Disease Control and Prevention guidelines. A total of 30 age- and sex-matched healthy donors were recruited as controls. Patients who met any one of the following criteria were classified as severe: ARDS, shock, multiorgan failure, requiring ICU admission, or mechanical ventilation for medical reasons. This study was conducted in accordance with the amended Declaration of Helsinki, and the protocol was approved by the Ethics Committee of the Binzhou Medical University Hospital, and was registered in the Clinical Trials Register (ChiCTR2000040921). Informed consent was obtained from patients (or their caregivers) and healthy controls before enrollment.

### Data and blood sample collection

Demographic characteristics, including sex, age, comorbidity, and symptoms at onset of illness, were recorded on admission. The Sequential Organ Failure Assessment (SOFA) score, and the Acute Physiology, Age, Chronic Health Evaluation II (APACHE II) score were calculated to assess illness severity. Clinical outcomes were assessed according to the 28-day mortality rate.

On the first medical diagnosis, a venous blood sample was collected in a K2 EDTA tube. Venous blood samples were collected from the patients and centrifuged at 4000×g for 10 min, and plasma was collected and stored at −80 °C until analysis. The lactate level, blood routine index, hepatic and renal function, and coagulation/fibrinolysis markers were analyzed in a standardized laboratory. Plasma levels of tumor necrosis factor-α (TNF-α) (Besançon cedex, France, 950.090.096, Diaclone), interleukin-6 (IL-6) (Besançon cedex, France, 950.030.096, Diaclone) and IL-10 (Besançon cedex, France, 950.060.096, Diaclone), SDC-1 (Besançon cedex, France, 950.640.096, Diaclone), HA (Minneapolis, USA, DHYAL0, R&D Systems), and HS (San Diego, USA, MBS285287, MyBioSource,) were measured using an enzyme-linked immunosorbent assay kit according to the manufacturer’s instructions.

### Statistical analysis

All categorical variables are expressed as numbers and percentages. Between*–*group comparisons of frequencies were analyzed using the chi*–*square test. Summary statistics for normally distributed continuous variables are presented as mean ± standard deviation, and non–normally distributed continuous variables are presented as median and interquartile range. Differences between groups were tested for significance using either the non–parametric Mann–Whitney *U* test or an unpaired Student’s *t*-test for two groups and the Kruskal–Wallis analysis of variance for multiple groups. Correlations were analyzed by the Spearman rank correlation test. The efficacy of each parameter in predicting the diagnostic power of each marker is expressed as the area under curve (AUC) using the receiver operating characteristic (ROC) analysis. Kaplan–Meier analysis was performed to compare the outcome of groups according to the SDC-1 cut–off. Survival curves were compared using a log–rank test. For all tests, a *P*-value of less than 0.05 was considered statistically significant. Statistical analyses were conducted using IBM SPSS 25.0.

## Results

### Patient characteristics

**Figure 1:**
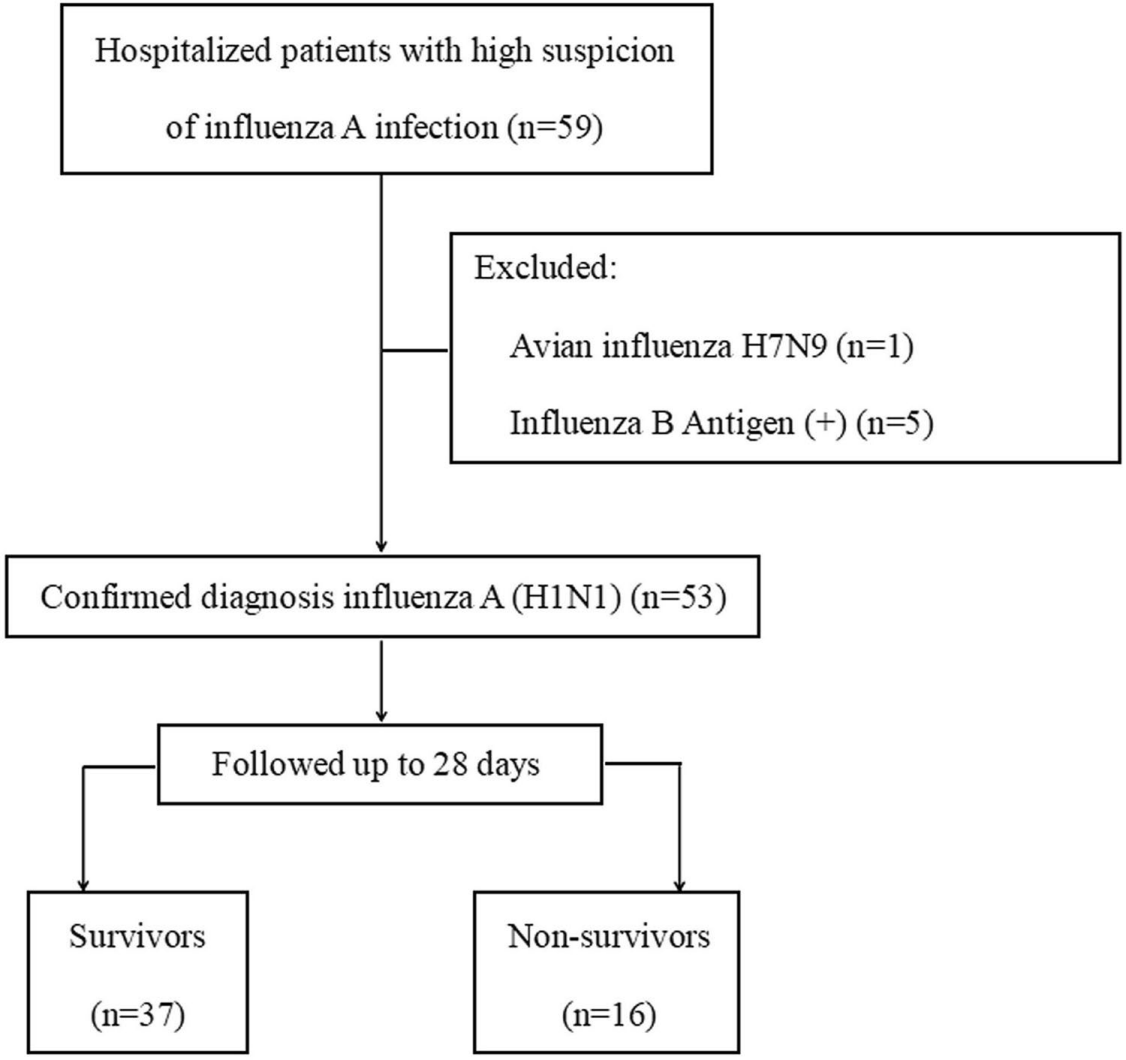
Flowchart of the study.

We initially screened 59 hospitalized patients with a high suspicion of influenza A infection (Fig. 1). We excluded one patient with avian influenza H7N9, and five patients with influenza B antigen (+). Thus, we enrolled 53 patients with a positive PCR test for influenza A (H1N1) and 30 healthy controls. The 30 healthy controls comprised 15 men and 15 women with a median age of 59 years. The 53 patients with influenza A (H1N1) comprised 20 men (37.73%) and 33 women (62.26%) with a median age of 57 years. Using the program for diagnosis and treatment of influenza A (H1N1), H1N1-infected patients were divided into two different groups based on clinical severity: the mild group (*n* = 15) and the severe group (*n* = 38). The patient characteristics are presented in Table 1.

**Table 1:** Characteristics and symptoms of patients infected with Influenza A (H1N1).

Characteristics	Control subjects (*N* = 30)	Total Influenza A (H1N1) (*N* = 53)	Mild Influenza A (H1N1) (*N* = 15)	Severe Influenza A (H1N1) (*N* = 38)	*P* Value
Age, years (Median, P25-P75)	59 (53–63)	57 (47, 69)	56 (47, 76)	59 (44, 69)	0.96
Sex (Male/Female)	15/15	20/33	7/8	13/25	0.393
Smoker (*n*, %)	8, 26.67%	**20, 37.73%**	**10, 66.67%**	**10, 26.32%**^**a**^	**0.006**
Lactate (mmol/L)	–	**1.5 (1.0, 2.7)**	**1.1 (0.9, 1.5)**	**2.1 (0.9, 3.1)**^**b**^	**0.012**
≥ 2 (*n*, %)		**21, 39.62%**	**1, 6.67%**	**20, 52.63%**^**a**^	**0.002**
APACHE II score	–	**11 (8, 15)**	**8 (5, 9)**	**14 (10,18)**^**b**^	** < 0.001**
SOFA score	–	**5 (2, 7)**	**2 (1, 2)**	**6 (4, 8)**^**b**^	** < 0.001**
Comorbidity (*n*, %)	–				
Hypertension		15, 28.30%	2, 13.33%	13, 34.21%	0.129
Cardiovascular disease		6, 11.32%	2, 13.33%	4, 10.53%	0.771
Diabetes		8, 15.09%	1, 6.67%	7, 18.42%	0.282
Cerebrovascular disease		3, 5.66%	1, 6.67%	2, 5.26%	0.842
Chronic obstructive		3, 5.66%	1, 6.67%	1, 2.63%	0.487
Asthma		2, 3.77%	**2, 13.33%**	**0, 0**^**a**^	**0.022**
Chronic hepatitis		2, 3.77%	0, 0	2, 5.26%	0.365
Malignancy		3, 5.66%	2, 13.33%	1, 2.63%	0.144
Without		9, 16.98%	1, 6.67%	8, 21.05%	0.209
Symptoms at onset of illness	–				
Highest temperature, ℃		38.5 (38.0, 39.1)	38.6 (37.9, 39.1)	38.5 (38.0, 39.1)	0.993
< 37.3 (*n*, %)		5, 9.43%	2, 13.33%	3, 7.89%	0.542
37.3–38 (*n*, %)		13, 24.53%	3, 20.00%	10, 26.31%	0.630
38.1–39 (*n*, %)		19, 35.85%	6, 40.00%	13, 34.21%	0.692
> 39 (*n*, %)		16, 30.19%	4, 26.67%	12, 31.58%	0.726
Cough (*n*, %)		42, 79.25%	11, 73.33%	31, 81.58%	0.505
Sputum production (*n*, %)		33, 62.26%	9, 60.00%	24, 41.38%	0.831
Oppression (*n*, %)		22, 41.51%	9, 60.00%	13, 34.21%	0.086
Chilly (*n*, %)		14, 26.41%	4, 26.67%	10, 26.32%	0.979
Fatigue (*n*, %)		8, 15.09%	2, 13.33%	6, 11.32%	0.822
Myalgia (*n*, %)		5, 9.43%	2, 13.33%	3, 5.17%	0.542
Headache (*n*, %)		3, 5.66%	2, 13.33%	1, 2.63%	0.129
Chest pain (*n*, %)		**4, 7.55%**	**3, 20.00%**	**1, 2.63%**^**a**^	**0.031**
Pharyngalgia (*n*, %)		3, 6.66%	1, 6.67%	2, 3.45%	0.842
Anorexia (*n*, %)		5, 9.43%	0, 0	5, 8.62%	0.134
Vomiting (*n*, %)		1, 1.88%	0, 0	1, 1.72%	0.526
Dizziness (*n*, %)		3, 5.66%	1, 6.67%	2, 3.45%	0.842
P/F ratio		**223 (112, 311)**	**328 (297, 366)**	**164 (76, 205)**^**b**^	** < 0.001**
During hospitalization stay Ventilatory support (*n*, %)					
Nasal catheters for oxygen			10, 66.67%	2, 5.26%	
Non invasive ventilation			0, 0	6, 15.79%	
High flow nasal cannula			0, 0	8, 21.05%	
Invasive mechanical ventilation			0, 0	22, 57.89%	
Steroid treatment		20, 37.74%	6, 40.00%	14, 36.84%	0.831
Hospital length of stay, days		11 (6, 18)	**8 (6, 14)**	**14 (9, 21)**^**a**^	**0.044**
ICU length of stay, days			-	9 (4, 13)	
28-day mortality, (*n*, %)		16, 30.19%	-	16, 42.11%	

Data are expressed as median (inter-quartile range), mean ± SD, or number (%). Abbreviations: APACHE, Acute Physiology, Age, Chronic Health Evaluation; SOFA, Sequential organ failure assessment; P/F, PaO_2_/FiO_2_. a, vs. Mild group, χ2 tests; b, vs. Mild group, Mann–Whitney *U* test. Kruskal–Wallis test was used between the age of control, mild and severe groups; χ2 test was used between the male to female ratio of control, mild and severe groups. –, This data were not collected initially.

The most common clinical features at illness onset included a fever of > 38℃ (*n* = 35, 66.04%), cough (*n* = 42, 79.25%), sputum production (*n* = 33, 62.26%), oppression, (*n* = 22, 41.51%) and chilly (*n* = 14, 26.41%). Less common symptoms were myalgia, chest pain, pharyngalgia, anorexia, etc. The most common comorbidities were hypertension (*n* = 15, 28.30%), cardiovascular disease (*n* = 6, 11.32%), and diabetes (*n* = 8, 15.09%); however, 9 patients presented without comorbidity. Two patients in the mild group presented with asthma; this comorbidity was not present among patients in the severe group. The P/F ratio tended to be lower in the severe group than in the mild group. Invasive mechanical ventilation was required in > 50% of the patients in the severe group. There was no statistically significant difference in the number of patients taking steroid treatment between the two groups. The length of hospital stay was significantly longer in the severe group than in the mild group. All participants were followed-up for 28 days, at which point there were 37 survivors and 16 non-survivors.

### Laboratory records and inflammation markers

The median white blood cell count in both the mild and severe groups were within the normal range; however, the count was significantly higher in the severe group; 50% of the patients in the severe group had white blood cell counts of > 10 × 10^9^/L. The lymphocyte count and percentage were significantly lower in the severe group than in the mild group. The alanine aminotransferase (ALT) levels were higher than 35 U/L in 17 patients in the severe group and four in the mild group. The aspartate aminotransferase (AST) levels were higher than 40 U/L in 24 patients in the severe group and one in the mild group. Concurrently, the proportion of patients with increased AST was significantly higher in the severe group than in the mild group (24/38 [63.16%] vs. 1/15 [6.67%], *P* < 0.001). The lactate dehydrogenase and procalcitonin levels were significantly higher in the severe group than in the mild group. However, the albumin level was significantly lower in the severe group than in the mild group. The severe group also exhibited marked increases in TNF-α, IL-6, and IL-10 levels relative to the mild group. The laboratory findings and inflammation markers of patients with influenza A (H1N1) are presented in Table 2.

**Table 2:** Laboratory records and inflammation markers of patients infected with Influenza A (H1N1) on admission.

Characteristics	Control subjects (*N* = 30)	Mild influenza A (H1N1) (*N* = 15)	Severe influenza A (H1N1) (*N* = 38)	*P* value
White blood cell count × 10^9^/L (4–10)	**5.9 (5.2, 6.5)**	**7.2 (5.5, 9.0)**	**10.3 (6.0, 16.1)**^**b**^	**0.024**
< 4	0	2, 13.33%	2, 5.26%	0.044
4–10	30	11, 73.33%	17, 44.74%	…
> 10	0	2, 13.33%	19, 50.00%	…
Lymphocyte count × 10^9^/L (1–3)	**1.8 (1.4, 2.3)**	**0.9 (0.6, 1.6)**	**0.6 (0.5, 0.9)**^**b**^	**0.042**
≤ 0.5	0	**3, 20.00%**	**16, 42.11%**^**a**^	** < 0.001**
0.5- 1.0	0	7, 46.67%	15, 39.47%	…
≥ 1.0	30	5, 33.33%	7, 18.42%	…
Lymphocyte percentage (%) (20–40)	31.8 (24.7 36.4)	**14.5 (8.6, 22.4)**	**7.2 (4.6, 12.4)**^**b**^	**0.017**
CD4 + T Lymphocytes (%) (30.1–44.4)	–	41.4 ± 8.2	36.4 ± 11.8	0.244
CD8 + T Lymphocytes (%) (20.7–29.4)	–	22.2 ± 5.3	23.2 ± 8.0	0.733
CD4/CD8 (1.02–1.94)	–	1.77 (1.40, 2.66)	1.71 (0.92, 2.41)	0.569
Platelet count, × 10^9^/L (100–300)	256.0 (143.5, 284.0)	233.5 (173.0, 291.0)	154.0 (105.0, 255.0)	0.508
D-dimer, μg/mL (0–0.5)	–	**0.32 (0.22, 0.68)**	**3.75 (1.50, 13.27)**^**b**^	** < 0.001**
Albumin, g/L (40–55)	44.8 ± 3.1	**38.0 ± 4.8**	**27.6 ± 4.0**^**c**^	** < 0.001**
ALT, U/L (9–35)	17.4 (13.2, 24.6)	**18.5 (16.4, 39.9)**	**34.3 (20.5, 56.1)**^**b**^	** < 0.001**
≤ 35	30	11	21	0.226
> 35	0	4	17	…
AST, U/L (15–40)	21.1 (18.9, 26.8)	**23.1 (17.8, 27.0)**	**47.8 (34.8, 82.5)**^**b**^	**0.033**
≤ 40	30	**14**	**14**^**a**^	** < 0.001**
> 40	0	**1**	**24**	**…**
Creatinine, μmol/L (0–135)	45.9 (38.1, 56.2)	60.3 (43.6, 66.9)	59.8 (47.3, 91.3)	0.622
LDH, U/L (0–430.6)	194.6 (165.3, 223.6)	**223.9 (193.9, 239.5)**	**635.7 (375.6, 864.4)**^**b**^	** < 0.001**
CK, U/L (25–200)	67.3 (47.1, 97.6)	65.9 (45.1, 130.5)	86.3 (42.8, 272.3)	0.365
PCT, μg/L (0–0.5)	–	**0.06 (0.04, 0.13)**	**0.68 (0.40, 4.80)**^**b**^	**0.001**
Inflammation markers				
TNF-α (pg/ml)	3.7 (1.3, 5.6)	**8.4 (6.7, 13.3)**^**d**^	**13.3 (11.7, 23.2)**^**b, d**^	**0.001**
IL-6 (pg/ml)	1.9 (0.5, 5.9)	**3.4 (2.6, 5.5)**^**d**^	**71.2 (16.6, 182.0) b, d**	** < 0.001**
IL-10 (pg/ml)	5.9 (4.7, 6.5)	**9.1 (6.6, 15.9)**^**d**^	**22.4 (12.3, 52.6)**^**b, d**^	** < 0.001**

Data are expressed as median (inter-quartile range), mean ± SD, or number (%). Abbreviations: ALT, Alanine aminotransferase; AST, Aspartate aminotransferase; LDH, Lactate dehydrogenase; CK, Creatine kinase; PCT, Procalcitonin; TNF, tumor necrosis factor; IL-6, interleukin-6; IL-10, interleukin-10. ^a^, vs. Mild group, χ2 tests; ^b^, vs. Mild group, Mann*–*Whitney *U* test; ^c^, unpaired Student *t* test. d, vs. Control group, Kruskal*–*Wallis test. –, This data were not collected initially.

### Glycocalyx component levels and biomarker accuracy

**Figure 2:**
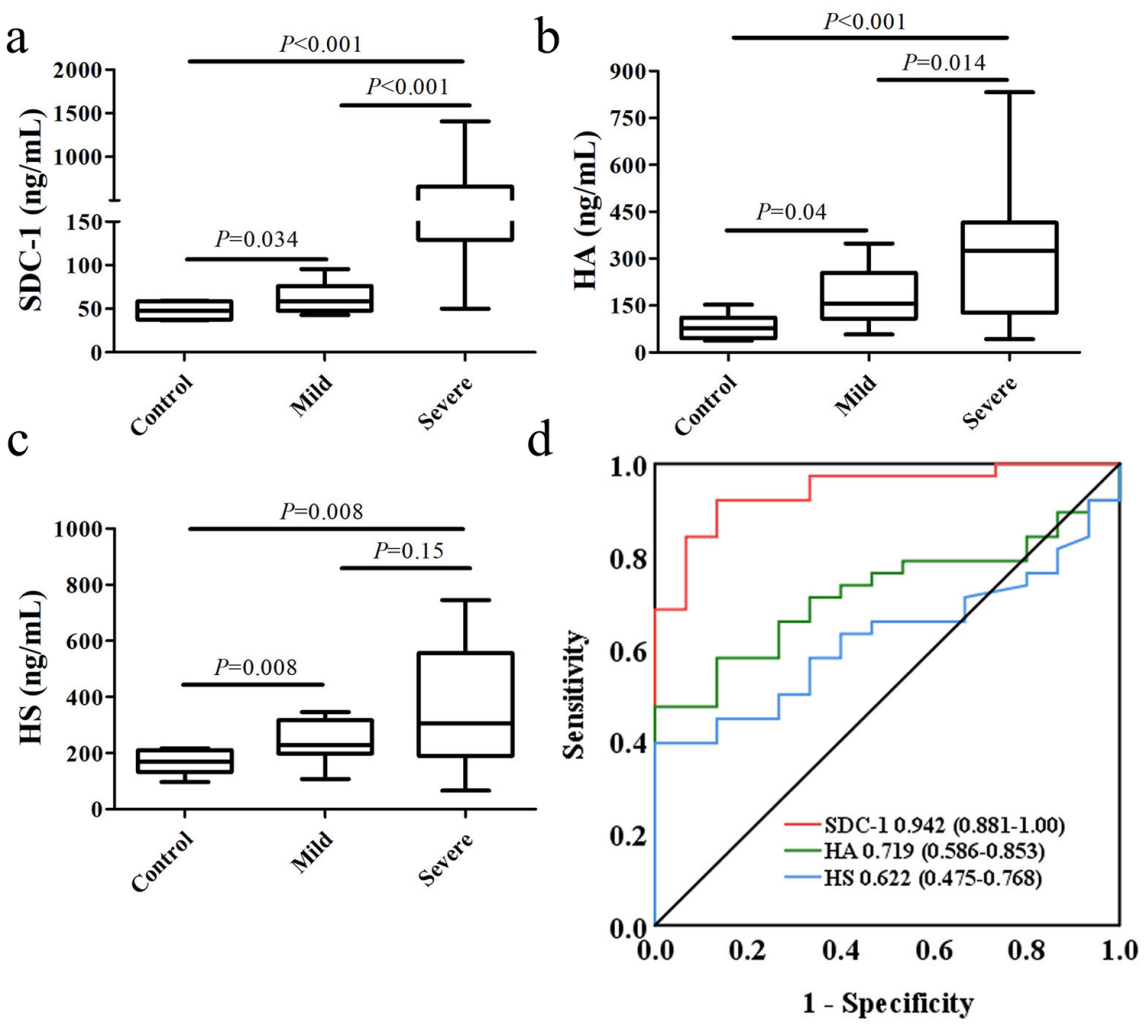
Plasma levels of glycocalyx components and ROC curves for the prediction of severe influenza A (H1N1). Plasma SDC-1 (**a**), HA (**b**), and HS (**c**) levels were significantly elevated in septic shock patients compared with those in sepsis patients. ROC curves (**d**) for the prediction of septic shock. AUC (95% confidence interval). The red rulers in figures A and B represent the median with range. The red rulers in figure C represent the mean with SD. Three-group comparisons of frequencies were analyzed by Kruskal*–*Wallis test. SDC-1, syndecan-1; HS, heparan sulfate; HA, hyaluronan.

The plasma levels of SDC-1 (257.9 [142.1–658.8] vs. 58.6 [49.4–75.9] ng/ml), HA (334.4 [159.3–413.9] vs. 156.6 [116.1–230.5] ng/ml), and HS (305.1 [193.9–516.9] vs. 228.8 [198.9–312.4] ng/ml) were significantly higher in the severe group than in the mild group (Fig. 2). We conducted a ROC analysis to determine the utility of glycocalyx components as markers of severe disease. The diagnostic accuracy for the identification of patients progressing to severe influenza A (H1N1) was highest for SDC-1, with an AUC of 0.942 (95% CI: 0.881–1.000; *P* < 0.001; Fig. 2d). At a cutoff point of > 81.0 ng/mL, SDC-1 had a sensitivity of 92.1% and specificity of 86.7% for diagnosing severe influenza A (H1N1).

### SDC-1 levels and disease severity

To identify the clinical relevance of endothelial glycocalyx components, we analyzed the correlation between the following variables: SDC-1, HA, and HS levels; SOFA score; APACHE II score; and various biochemical parameters including lactate, albumin, inflammatory markers (TNF-α, IL-6, and IL-10), and global hemostatic markers (platelet count and D-dimer).

The SOFA and APACHE II scores indicate organ dysfunction and disease severity in critically ill patients independent of the underlying disease, and are therefore commonly used to predict mortality in ICU patients. Spearman’s correlation analysis showed that the SDC-1 and HA levels were significantly correlated with the APACHE II (r = 0.526, *P* < 0.001; r = 0.439, *P* = 0.001) and SOFA scores (r = 0.515, *P* < 0.001; r = 0.409, *P* = 0.002) (Table 3, Supplementary Fig. 1). High lactate level is traditionally considered as a marker of tissue hypoxia, and can be considered as a warning signal for organ dysfunction^19^. Among the glycocalyx components, lactate levels were significantly positively correlated with SDC-1 (r = 0.392, *P* = 0.004) and HA (r = 0.372, *P* = 0.006) levels (Table 3, Supplementary Fig. 1). SDC-1 and HA were negatively correlated with the albumin levels (Table 3, Supplementary Fig. 2). The platelet count and D-dimer concentration reflect coagulation function. SDC-1 and HA were negatively correlated with the platelet count and positively correlated with the D-dimer concentration (Table 3, Supplementary Fig. 3). We also observed that SDC-1 and HA were positively correlated with IL-6 and IL-10 levels (Table 3, Supplementary Fig. 4).

**Table 3:** Correlations between glycocalyx components levels and various clinical parameters.

Characteristics	SDC-1		HA		HS	
	r	*P* Value	r	*P* Value	r	*P* Value
APACHE II score	**0.526**	** < 0.001**	0.439	0.001	0.047	0.739
**SOFA score**	**0.515**	** < 0.001**	0.409	0.002	0.063	0.652
Lactate	0.392	0.004	0.372	0.006	0.198	0.155
**Albumin**	-**0.639**	** < 0.001**	-0.399	0.003	0.03	0.831
Platelet	-0.313	0.017	**-0.473**	** < 0.001**	0.071	0.616
**D-dimer**	**0.593**	** < 0.001**	0.299	0.031	0.209	0.138
TNF-α (pg/ml)	0.276	0.046	0.245	0.077	-0.13	0.352
**IL-6 (pg/ml)**	**0.63**	** < 0.001**	**0.549**	** < 0.001**	0.20	0.15
**IL-10 (pg/ml)**	**0.50**	** < 0.001**	**0.622**	** < 0.001**	0.152	0.279

APACHE, Acute Physiology, Age, Chronic Health Evaluation; SOFA, Sequential organ failure assessment; TNF, tumor necrosis factor; IL-6, interleukin-6; IL-10, interleukin-10; SDC-1, syndecan-1; HS, heparan sulfate; HA, hyaluronan. r, Spearman's correlation coefficient.

### Predictors of 28-day mortality

**Figure 3:**
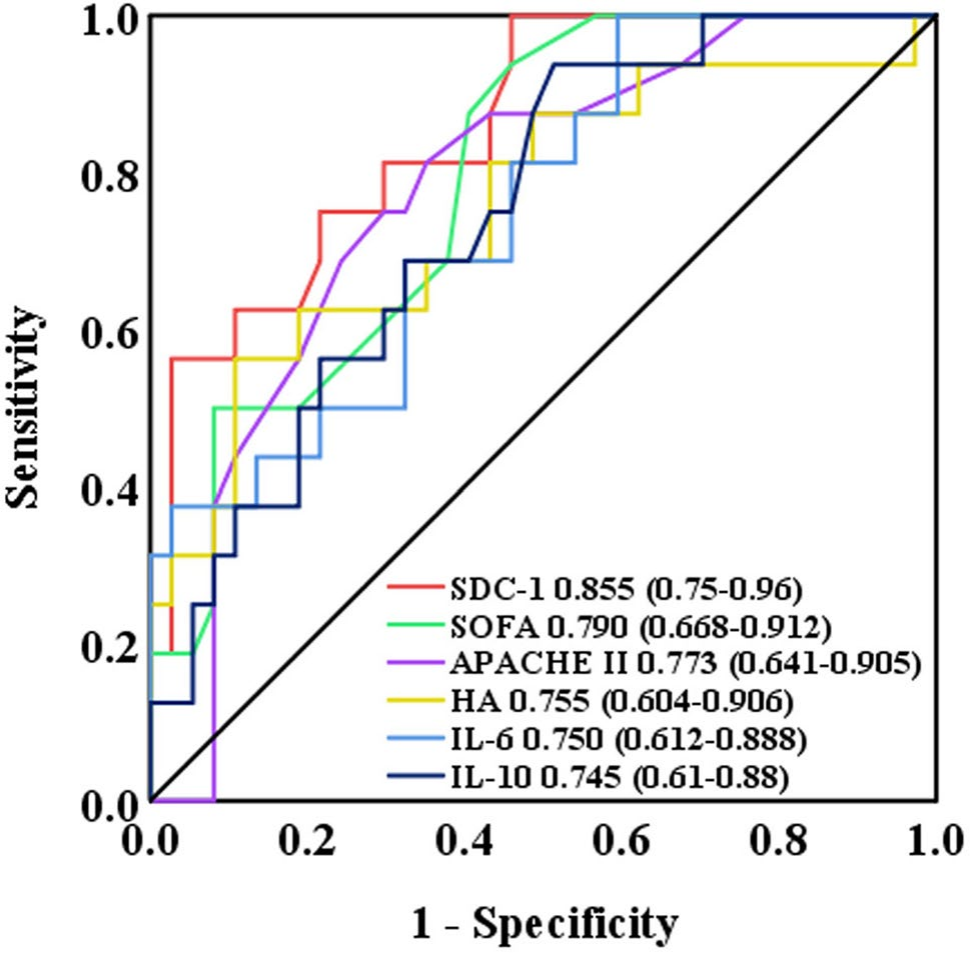
ROC curves for the prediction of 28-day mortality in patients with influenza A (H1N1).

**Figure 4:**
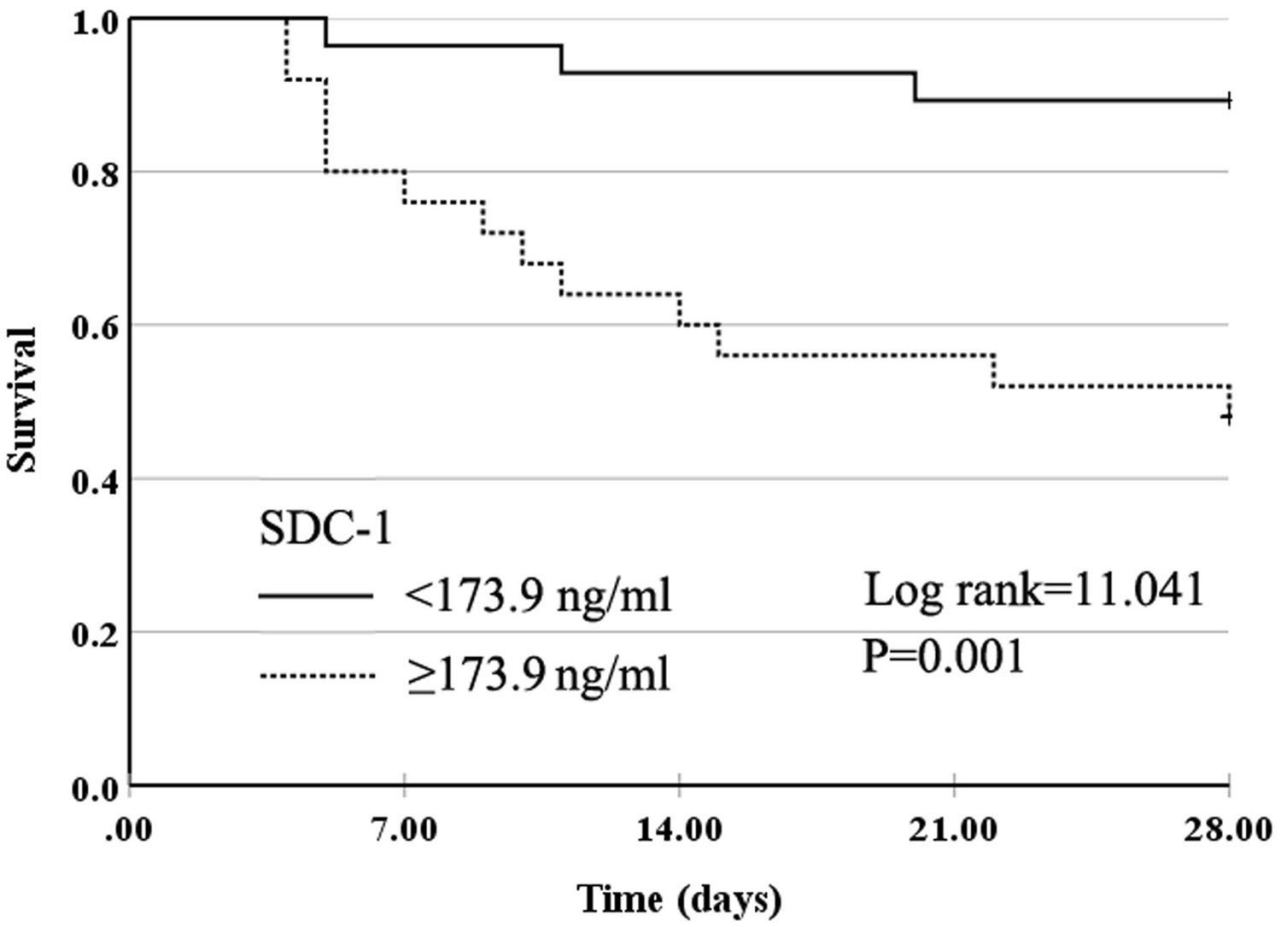
Kaplan*–*Meier survival estimates for all patients with influenza A (H1N1) infection according to the circulating levels of syndecan-1 (SDC-1, cut-off: 173.9 ng/ml).

All study participants were followed-up for 28 days and divided into the survivor and non-survivor groups (Table 4). The APACHE II and SOFA scores and lactate, D-dimer, IL-6, IL-10, SDC-1, and HA levels were significantly higher in non-survivors than in survivors. The albumin level and lymphocyte percentage were significantly lower in non-survivors than in survivors. We constructed ROC curves to determine the sensitivity and specificity of the APACHE II and SOFA scores, laboratory records, inflammation markers, and endothelial glycocalyx markers, in order to predict 28-day mortality (Fig. 3). The AUC for the SDC-1 was 0.855 (95% CI 0.75–0.96), which was higher than that for other indicators. At a cutoff point of ≥ 173.9 ng/ml, SDC-1 provided a specificity of 81.3% and a sensitivity of 70.3% for predicting 28-day mortality. Kaplan–Meier survival analyses are shown in Fig. 4. Patients with plasma SDC-1 ≥ 173.9 ng/ml had a significantly higher 28-day mortality estimate than patients with plasma SDC-1 < 173.9 ng/ml (*P* = 0.001).

**Table 4:** The APACHE II and SOFA scores, laboratory records, inflammation markers, and endothelial glycocalyx markers in the survivors and non-survivors (28-day death) on the admission.

Characteristics	Survivor (*N* = 37)	Non-survivor (*N* = 16)	*P* Value
APACHE II score	**9 (7, 13)**	**15 (14, 18)**^**b**^	** < 0.001**
SOFA score	**3 (2, 6)**	**7 (5, 9)**^**b**^	**0.001**
Lactate	**1.2 (0.9, 1.6)**	**2.2 (1.5, 3.5)**^**b**^	**0.001**
Lymphocyte count, × 109/L	**0.9 (0.5, 1.4)**	**0.6 (0.3, 0.9)**^**b**^	**0.048**
Lymphocyte percentage	**11.5 (7.1, 18.6)**	**5.4 (4.2, 7.8)**^**b**^	**0.002**
Platelet	210.5 (144.0, 272.0)	136.5 (85.5, 230.0)^b^	0.075
D-dimer (μg/mL)	**1.52 (0.49, 3.72)**	**6.26 (2.27, 22.1)**^**b**^	**0.012**
Albumin, g/L	**32.2 ± 6.6**	**27.2 ± 4.2**^**c**^	**0.007**
Inflammation markers			
TNF-α (pg/ml)	11.6 (8.4, 21.6)	14.1 (10.0, 35.7)^b^	0.322
IL-6 (pg/ml)	**15.4 (4.5, 96.7)**	**82.5 (15.8, 376.1)**^**b**^	**0.046**
IL-10 (pg/ml)	**11.27 (7.0, 23.1)**	**30.1 (14.1, 57.6)**^**b**^	**0.036**
Endothelial glycocalyx markers			
SDC-1 (ng/ml)	**117.7 (65.3, 258.5)**	**576.3 (204.7, 755.3)**^**b**^	**0.003**
HA (ng/ml)	**190.4 (108.4, 348.2)**	**400.5 (213.1, 431.3)**^**b**^	**0.02**
HS (ng/ml)	**233.8 (178.8, 331.7)**	**419.7 (201.4, 1446.6)**^**b**^	**0.034**

Data are expressed as median (inter-quartile range), mean ± SD, or number (%). Abbreviations: APACHE, Acute Physiology, Age, Chronic Health Evaluation; SOFA, Sequential organ failure assessment; TNF, tumor necrosis factor; IL-6, interleukin-6; IL-10, interleukin-10; SDC-1, syndecan-1; HS, heparan sulfate; HA, hyaluronan. ^b^, vs. Mild group, Mann*–*Whitney *U* test; ^c^, unpaired Student *t* test.

## Discussion

In this study, we aimed to determine whether the plasma levels of glycocalyx components are indicative of influenza A (H1N1) severity. We found that SDC-1, HS, and HA levels were significantly higher in patients with severe influenza A (H1N1) than in patients with mild disease. In addition, the severity of endothelial glycocalyx shedding was closely associated with disease severity, and facilitated the identification of patients with severe influenza A (H1N1). Furthermore, we found that the level of SDC-1 was a good predictor for 28-day mortality among patients with influenza A (H1N1). To our knowledge, this study is the first to compare plasma glycocalyx components in patients with mild and severe influenza A (H1N1).

Pandemic 2009 influenza A (H1N1) viral infections continue to be a public health threat^20^. Influenza A (H1N1)-infected patients most often have a mild clinical course; but the mortality rate is higher in severe cases. Naudion et al*.*^21^ reported that nineteen patients (9.1%) died in the hospital during the study period. Lobo et al*.*^22^ reported that the mortality rate for the patients with severe acute respiratory infection in the influenza A (H1N1) positive was 29.5%. In our study, the 28-day mortality rate of severe H1N1 patients was 42.1%. Furthermore, we found that approximately 21% of patients with severe H1N1 had no underlying disease. Several studies have found that both young subjects and adults may develop a severe clinical course of H1N1 infection without having any known risk factors^23,24^. The underlying pathogenic mechanisms have not been fully elucidated. In the ICU, patients with influenza A (H1N1) often present with viral pneumonia, severe hypoxemic respiratory failure, and ARDS. Currently, treatment for patients with severe influenza A (H1N1) is limited to antiviral drugs and symptomatic treatment, and which mainly consists of the optimization of oxygen supply and transfer through ventilation.

The severity of influenza A has been attributed to a systemic and inflammatory process that damages not only the lungs, but also multiple organs, including the central nervous system and cardiovascular system^25,26^. In our study, we found that patients with severe influenza A (H1N1) had increased levels of D-dimer, indicating coagulation disorders. The multiple organ damage caused by influenza A may be due to inflammation and vascular endothelial cell injury^9^. The cytokines storms caused by influenza have been associated with pro-inflammatory response disorder, which may lead to significant pathological consequences and poor prognosis^6,27,28^. The levels of pro-inflammatory cytokines are closely related to outcomes in patients with severe influenza^27–29^. This is consistent with our observation of significantly higher levels of inflammatory cytokines (IL-6 and IL-10) in non-survivors compared to survivors.

The levels of endothelial glycocalyx components, including SDC-1, HS, and HA, are useful biomarkers for sepsis^15,16^, ARDS^13,17^, and COVID-19^14,30^. Further, the APACHE II and SOFA scores may be used to assess the risk of death in critically ill patients, regardless of the primary disease. We found that plasma levels of SDC-1 and HA were significantly higher in patients with severe H1N1 than in patients with mild H1N1, and were positively correlated with the APACHE II and SOFA scores. Furthermore, at a cutoff point of ≥ 173.9 ng/ml, SDC-1 showed a specificity of 81.3% and sensitivity of 70.3% for predicting 28-day mortality. The plasma SDC-1 levels ≥ 173.9 ng/ml were associated with mortality in hospitalized patients with influenza A (H1N1) virus infection.

A few of the pathophysiological features of severe influenza pneumonia include small vessel thrombosis, hemorrhage, and diffuse alveolar damage^31^. The surface of vascular endothelium is coated with glycocalyx, which is extremely important for the maintenance of antithrombogenicity in the vascular lumen^32^. Disturbance of the glycocalyx is often due to the increased expression and release of proteinases (matrix metalloproteinases) and glycosidases (e.g., hyaluronidases and heparinase)^33^. Aggregation of activated platelets within the pulmonary microvasculature promotes lung inflammation and injury^34^; shedding of the glycocalyx results in promoted platelet aggregation and adhesion to the endothelium and subsequent thrombi formation^35^. Chappell et al*.*^36^ reported that protection from glycocalyx shedding reduces platelet adhesion in ischemia/reperfusion injury. We previously found that an increasing level of SDC-1 can be used as a biomarker for predicting diffuse intravascular coagulation (DIC) development with sepsis^11^ and that non-anticoagulant heparin can improve coagulation by inhibiting the activity of heparinase and reducing the shedding of glycocalyx in sepsis rats^37^. It is well known that a sharp increase in D-dimer, a secondary fibrinolytic specific molecular marker, typically indicates the existence of a thrombus. Wang et al*.*^38^ reported that the significantly increased D-dimer and corresponding hypoxemia indicated to the probability of pulmonary microthrombus. Several studies have shown that elevated plasma D-dimer is a prognostic factor for adverse outcomes in COVID-19^39^, influenza A(H1N1)^38^, and chronic obstructive pulmonary disease (COPD)^40^. In this study, the AUCs predicted by SDC-1 for severe influenza A (H1N1) and 28-day mortality were 0.942 (95% CI 0.881–1.00), 0.855 (95% CI 0.75–0.96), which were higher than that for D-dimer 0.845 (95% CI 0.733–0.956), 0.791 (95% CI 0.654–0.927) (Supplementary Fig. 5). In conclusion, the destruction of the glycocalyx can lead to platelet aggregation and subsequent thrombi formation; SDC-1 was more reliable than D-dimer in predicting the prognosis of influenza A (H1N1).

Shedding of the glycocalyx results in pulmonary microvascular endothelial barrier disorder, mainly characterized by albumin exudation and tissue edema^10^. In this study, we observed a significant negative correlation (r = −0.639) between the plasma albumin level and SDC-1 level. Thus, we hypothesized that the shedding of the glycocalyx was associated with a decrease in blood albumin levels. Albumin has multiple biological activities including antioxidant effects^41^, and maintains vessel wall integrity. Although albumin has a net negative charge, its amphoteric nature promotes tight binding to the glycocalyx, with the net effects of reducing hydraulic conductivity across the vascular barrier, resisting glycocalyx degradation (i.e., protecting against shedding), and contributing to the maintenance of vascular integrity and normal capillary permeability^42,43^. Jacob et al*.*^44^ showed that 5% albumin prevented glycocalyx degradation more effectively than did HES (6% hydroxyethyl starch 130/0.4) and 0.9% normal saline in their animal heart model. We believe that the effectiveness of albumin administration in preventing glycocalyx degradation in severe influenza or ARDS needs to be further explored.

Our study has several limitations. First, this study was conducted at a single center and involved a relatively small number of patients with influenza A (H1N1); our findings require large-scale clinical validation in order to be generalized. Therefore, our data should be interpreted with caution. Second, the data presented here were based on single time-point measurements and were not consecutive, the dynamic change was unknown, and the predictive value needs further evaluation. Despite the above limitations, we believe that our study has yielded important and novel findings regarding the prediction of mortality in hospitalized patients with influenza A (H1N1).

## Conclusions

Plasma glycocalyx component levels are significantly higher in patients with severe influenza A (H1N1) than in mild cases. Specifically, SDC-1 and HA are closely correlated with APACHE II and SOFA scores, albumin, platelets, and D-dimer. Plasma SDC-1 levels of ≥ 173.9 ng/ml were associated with 28-day mortality in patients with influenza A (H1N1). Thus, increased plasma SDC-1 may be a biomarker of disease severity and could be useful for predicting 28-day mortality in patients with influenza A (H1N1).

## Ethics approval statement

This study was performed in accordance with the Declaration of Helsinki and approved by research ethics board of Binzhou Medical University Hospital. The study was registered in the Clinical Trials Register (ChiCTR2000040921).

## Supplementary Information

Supplementary Information.

## Data Availability

The data that support the findings of this study are available on request from the corresponding author. The data are not publicly available due to privacy or ethical restrictions.
