# Supplementary material for: Association between plasma glycocalyx component levels and poor prognosis in severe influenza type A (H1N1)
Source: Sci Rep. 2022 Jan 7;12:163. doi: 10.1038/s41598-021-04146-2 (PMC8741814; doi:10.1038/s41598-021-04146-2)

Association between plasma glycocalyx component levels and poor prognosis in severe influenza type A (H1N1)

Supplementary figure legends

Supplementary Fig. 1 Correlations between SDC-1, HA, and HS levels and the APACHE II score, SOFA score, and lactate levels. r, Spearman's correlation coefficient. SDC-1, syndecan-1; HS, heparan sulfate; HA, hyaluronan.

Supplementary Fig. 2 Correlations between SDC-1, HA, and HS levels and the albumin levels. r, Spearman's correlation coefficient. SDC-1, syndecan-1; HS, heparan sulfate; HA, hyaluronan.

Supplementary Fig. 3 Correlations between SDC-1, HA, and HS levels and the platelet count and D-dimer levels. r, Spearman's correlation coefficient. SDC-1, syndecan-1; HS, heparan sulfate; HA, hyaluronan.

Supplementary Fig. 4 Correlations between SDC-1, HA, and HS levels and the TNF-α, IL-6 and IL-10 levels. r, Spearman's correlation coefficient. SDC-1, syndecan-1; HS, heparan sulfate; HA, hyaluronan.

Supplementary Fig. 5 ROC curves for the prediction of severe influenza A (H1N1) (A) and 28-day mortality in patients with influenza A (H1N1) (B).

Supplementary Fig. 1

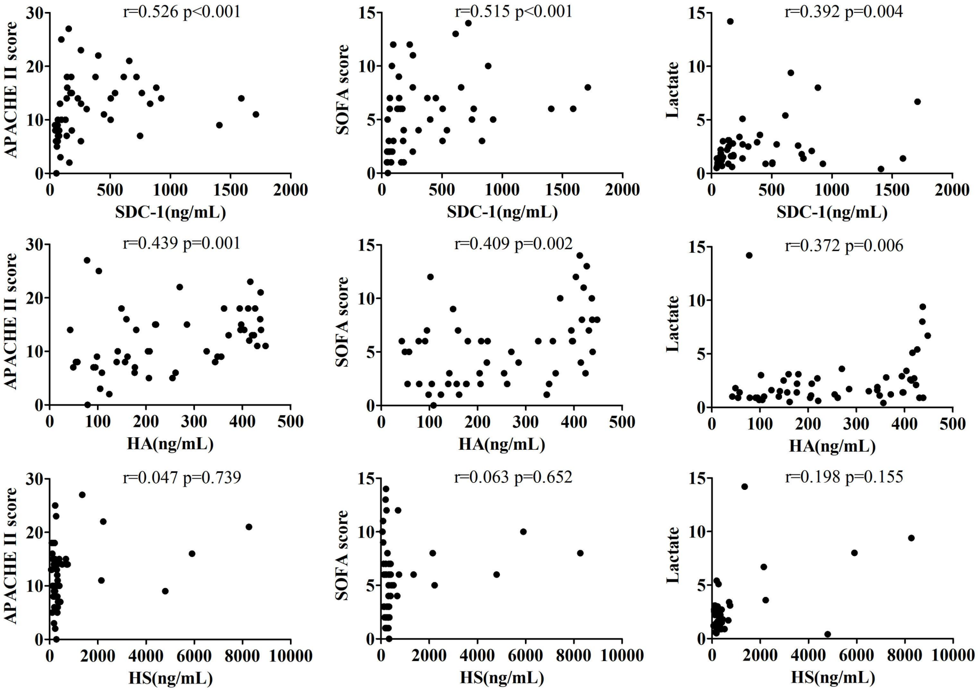

Supplementary Fig. 2

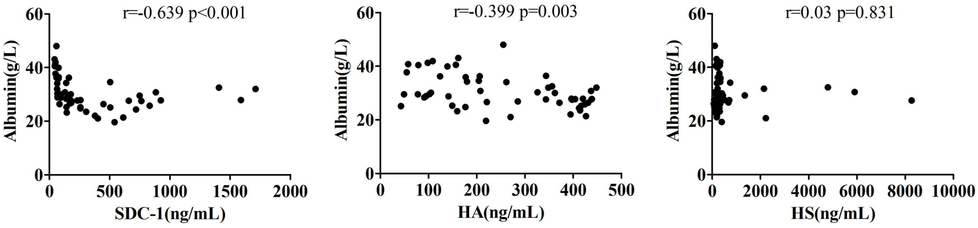

Supplementary Fig. 3

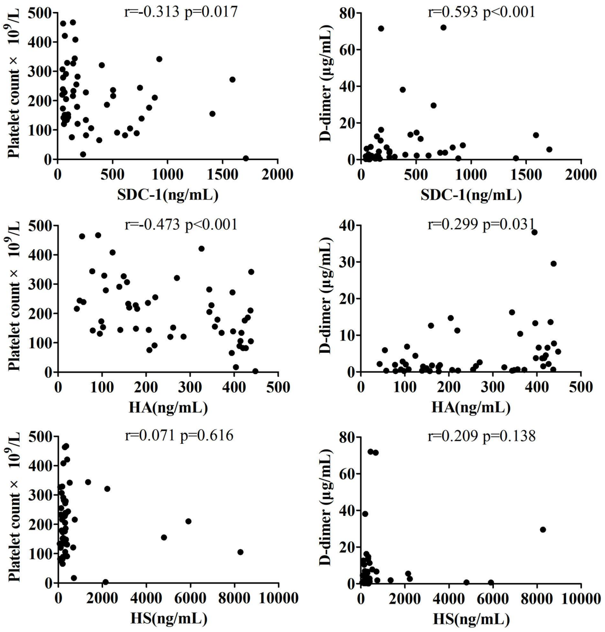

Supplementary Fig. 4

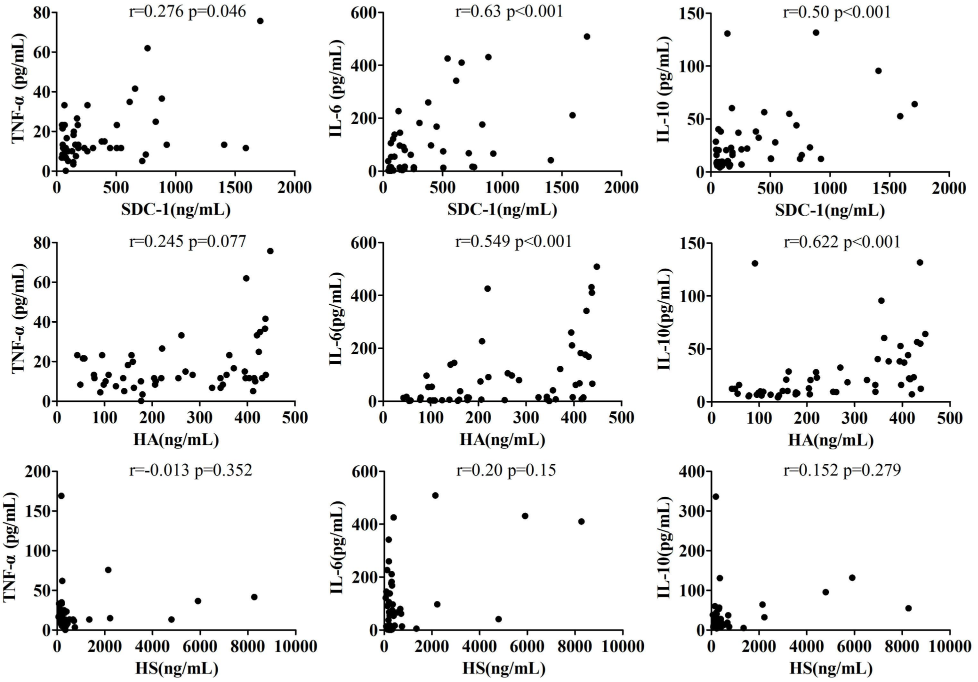

Supplementary Fig. 5

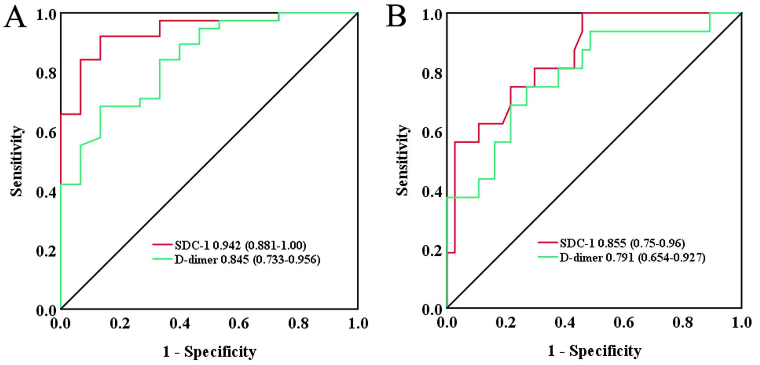

Supplement: Supplementary file 1 — Supplementary Information. [file 41598_2021_4146_MOESM1_ESM.pdf]
